# Susceptibility of wild and colonized *Anopheles stephensi* to *Plasmodium vivax* infection

**DOI:** 10.1186/s12936-018-2343-0

**Published:** 2018-06-05

**Authors:** Ajeet Kumar Mohanty, Praveen Balabaskaran Nina, Shuvankar Ballav, Smita Vernekar, Sushma Parkar, Maria D’souza, Wenyun Zuo, Edwin Gomes, Laura Chery, Shripad Tuljapurkar, Neena Valecha, Pradipsinh K. Rathod, Ashwani Kumar

**Affiliations:** 1 National Institute of Malaria Research, Field Unit, Campal, Goa 403001 India; 2 Departments of Chemistry and Global Health, University of Washington, Seattle, WA 98195 USA; 3 Department of Epidemiology and Public Health, Central University of Tamil Nadu, Thiruvarur, Tamil Nadu 610005 India; 4 Goa Medical College and Hospital, Bambolim, Goa 403202 India; 5 Department of Biology, Stanford University, Stanford, CA 94305 USA; 6 National Institute of Malaria Research (ICMR), Sector 8, Dwarka, New Delhi 110077 India

## Abstract

**Background:**

As much as 80% of global *Plasmodium vivax* infections occur in South Asia and there is a shortage of direct studies on infectivity of *P. vivax* in *Anopheles stephensi*, the most common urban mosquito carrying human malaria. In this quest, the possible effects of laboratory colonization of mosquitoes on infectivity and development of *P. vivax* is of interest given that colonized mosquitoes can be genetically less divergent than the field population from which they originated.

**Methods:**

Patient-derived *P. vivax* infected blood was fed to age-matched wild and colonized *An. stephensi*. Such a comparison requires coordinated availability of same-age wild and colonized mosquito populations. Here, *P. vivax* infection are studied in colonized *An*. *stephensi* in their 66th–86th generation and fresh field-caught *An. stephensi*. Wild mosquitoes were caught as larvae and pupae and allowed to develop into adult mosquitoes in the insectary. Parasite development to oocyst and sporozoite stages were assessed on days 7/8 and 12/13, respectively.

**Results:**

While there were batch to batch variations in infectivity of individual patient-derived *P. vivax* samples, both wild and colonized *An. stephensi* were roughly equally susceptible to oocyst stage *Plasmodium* infection. At the level of sporozoite development, significantly more mosquitoes with sporozoite load of 4+ were seen in wild than in colonized populations.

## Background

*Plasmodium*-*Anopheles* interactions in malaria endemic research sites are widely studied using colonized mosquito populations [1–3]. Laboratory-adapted mosquitoes offer significant advantages in logistics, ease of maintenance, flexibility of scaling up and reproducibility of experimental infections. Many *Anopheles* species, especially the *Plasmodium* vectors *Anopheles gambiae*, *Anopheles dirus*, *Anopheles albimanus*, and *Anopheles darlingi* have been colonized and are used for experimental *Plasmodium* infections [2, 4–10]. However, it is also known that when colonized mosquitoes (and other insects) are maintained in the laboratory for generations, may not accurately reflect the genetic make-up of a wild population due to founder effects, inbreeding, genetic drift, and accumulation of traits that favour their survival in artificial breeding conditions [6, 11–14]. Colonized mosquito populations can also lose alleles that are required in the wild.

Studies in *Drosophila* show that the laboratory bred populations have lower fitness and are less adaptable compared to the outbred population from which they originated [15, 16]. Transcriptome analysis of wild and laboratory *An. gambiae* indicate substantive divergence, with elevated expression of genes involved in insecticide resistance, immunity and olfaction in wild mosquitoes, while metabolism and protein synthesis genes were expressed higher in colonized population [14]. In colonized *An. gambiae*, microsatellite DNA polymorphisms were lower compared to wild populations [17]. Similarly, in *An. albimanus* colonized for 20 years, significant genetic differentiation was found in the mitochondrial gene *cytb* gene between laboratory and field populations. Even after just 21 generations of colonization, low to moderate genetic diversity was observed in *An. darlingi* [18]. The interaction of *Plasmodium* with *Anopheles* in the wild must constantly evolve, and a successful vector-parasite association must depend on the ability of parasites to continuously adapt to the changing ecosystem. How mosquito genetic differentiation affects the susceptibility of colonized mosquitoes to *Plasmodium* infection is poorly understood. One way to study the effect of genetic diversity on vector-parasite interactions in wild and colonized mosquitoes would be to feed the same patient-derived *Plasmodium* blood to age-matched wild and laboratory mosquitoes, and track infection kinetics.

*Anopheles stephensi* is the major urban malaria vector in India and is a dominant vector in Goa, where the MESA-ICEMR (Malaria Evolution in South Asia-International Center of Excellence for Malaria Research) study site is located [19]. *Anopheles stephensi* was colonized from a single wild-caught female, and currently the colony is in its 86th generation. Infections in a high passaged *An. stephensi* line were compared with wild *An. stephensi*, after challenge with patient isolates of *P. vivax*. To get age matched wild mosquitoes, field-caught larvae and pupae of *An*. *stephensi* were allowed to develop into adult mosquitoes and immediately used for comparison with colonized *An. stephensi* of the same age (day 6 or 7). The study directly compares the susceptibility of wild and colonized *An. stephensi* when fed many different local patient isolates of *P. vivax* from South Asia.

## Methods

### Establishment and maintenance of pure colony of *Anopheles stephensi*

*Anopheles stephensi* larvae were collected by dipping technique from the curing waters, which are intended for curing the cement slabs in the construction sites in North Goa, India in January, 2012. The larvae were reared in plastic trays containing curing water collected from the breeding habitat and set in the laboratory at temperature 27 ± 2 °C, relative humidity 70 ± 5% with 12 h light/12 h dark photoperiod cycling. A pinch of Cerelac™ powder (about 60 mg) and fish food (1:1) mixture was given to the growing larvae once a day till they developed into pupae. The pupae were collected in plastic bowls containing 200 ml tap water and were kept inside a closed cage until the emergence of adults. Upon emergence, the adult mosquitoes were fed 10% glucose soaked in a cotton pad. Adult mosquitoes were identified using standard keys by trained experts. On day 5 post-emergence, 10 female mosquitoes were starved over-night (14–16 h) and then allowed to feed on human blood in a membrane feeder. A single fed female mosquito was kept inside the cage for oviposition, and its eggs were used for the continuous cyclic population of pure *An. stephensi* colony. Larvae hatched from these eggs were reared in reverse-osmosis purified water under the same laboratory conditions described above. The colonized mosquitoes used in these experiments were in their 66th–86th generation.

### Wild *Anopheles stephensi* larvae collection and maintenance

Larvae and pupae of wild *An. stephensi* were collected by dipping technique from the curing waters of natural breeding habitats in construction sites around the city of Ponda in Goa, India. Along with larvae and pupae, the surrounding water was also transferred to plastic bowls, and brought to MESA insectary at the NIMR Goa field station. In the laboratory, the third and fourth stage larvae were separated from the first and second, and were allowed to develop separately. The field collected pupae were kept in a separate 500 ml plastic bowl containing approximately 200 ml of tap water, and the bowl was kept in a cage for emergence. Temperature, humidity and feeding protocols were the same as described above for the pure colony. The emerged wild adult mosquitoes were species-verified using standard morphological keys by trained experts [20, 21].

### Approvals for the study

All necessary approvals for collecting blood from malaria patients and conducting the study were obtained from the Institutional Ethics Committee of Goa Medical College and Hospital (GMC), the University of Washington Institutional Review Board, NIH/NIAID Division of Microbiology and Infectious Disease (DMID), Health Ministry Screening Committee (HMSC) of the Government of India and by the Government of Goa Public Health Department.

### Collection of *Plasmodium vivax* infected blood

*Plasmodium vivax*-positive patients, identified by microscopy, were briefed at GMC about the study and informed consent was obtained from volunteers prior to blood collection. Approximately 5.5–6 ml of blood was drawn into acid dextrose vacutainer, and the vacutainer was immediately placed in a thermos flask maintained at 37 °C and transported to the MESA insectary at NIMR-Goa.

### Mosquito infection experiments

Six to seven-day old adult females of wild and colonized mosquito populations were used to compare their ability to support *P. vivax* infections. Based on the availability of the age-matched mosquitoes, 75–125 wild and colonized mosquitoes were selected for any single experiment. Each infection experiment used one patient’s blood to infect both wild and colonized mosquitoes. Over 30 separate experiments were conducted, some for monitoring the timing of infections and the rest to study the host-competence of wild versus laboratory-reared mosquitoes. The mosquitoes were securely kept in plastic cups covered by mesh netting. Since GMC blood collection clinical site is just 7 km from the MESA NIMR-Goa insectary, the freshly collected blood maintained at 37 °C in a water flask could be transferred within 60–90 min to feed the wild and colonized mosquitoes. Blood feeding was done by the standard membrane feeding assay (MFA) as described in earlier studies [2, 7]. Briefly, 2 ml of blood was added to a 5 cm water- jacketed membrane feeder positioned in the center of the plastic container containing mosquitoes, and fitted to a circulating water bath maintained at 37 °C. Mosquitoes were then allowed to feed for 90 min. After that, unfed mosquitoes were removed, and the plastic cup with fully-fed mosquitoes were kept in Percival incubators maintained at 27 °C ± 2 and 80% ± 2 relative humidity. Cotton pads soaked in 10% glucose were provided for subsequent days until the mosquitoes were dissected.

### Mosquito dissections, microscopy and parasite counting

Equal number of wild and colonized mosquitoes were dissected on days 7/8 post blood feeding for assessing oocyst load in the midgut. The oocysts were counted using a Carl Zeiss Axio Lab. A1 phase-contrast microscope at 5× and 10× magnification. Sporozoite load was assessed on days 12 and 13, and imaging of dissected salivary glands was done using Carl Zeiss Axio Lab. A1 phase contrast microscope at 40×. Sporozoite load was represented by gland index [4, 22, 23] and recorded as; 1+ for (0–10 sporozoites), 2+ for (10–100 sporozoites), 3+ for (101–1000 sporozoites), and 4+ for (> 1000 sporozoites). Parasitaemia in the patient blood was counted in thin smears by two trained technicians independently. For every smear, 100 fields were counted by the miller reticule technique [24]. The ratio of large reticule to small reticule was 4:1 (ImageJ software), and the reticule factor was 25.

### Statistics

Statistical analysis was performed using the GraphPad Prism 7.02 software. Assuming that the wild and the laboratory-maintained mosquito populations have the same SD, paired t test was used to determine p value (two tailed distribution, 95% confidence level). This was to assess the significance of differences seen in oocyst infection rate, average oocyst load, sporozoite infection rate and sporozoite load (4+) between wild and laboratory populations.

## Results

### Establishment of *Anopheles stephensi* colony

*Anopheles stephensi* adapts well to laboratory conditions and is easy to colonize. The colonized *An*. *stephensi* in the MESA-ICEMR study site, maintained for 4 years and currently in its 86th generation at the time of this writing, was derived from a single adult female caught from the field. The mosquitoes were fed with human blood, and the expected number of eggs, larvae, and pupae were obtained. Seasonality affected the amount of eggs produced, and time required for emergence of adult mosquitoes, with slow growth in the months between December and February. No alterations in lighting conditions or temperature in the laboratory were made for the growth and propagation of mosquitoes. For infection experiments, wild versus laboratory-reared 6–7 days old mosquitoes were used after starving them for 16–18 h.

### *Plasmodium vivax* oocyst production in wild versus laboratory-colonized *An. stephensi*

**Fig. 1:**
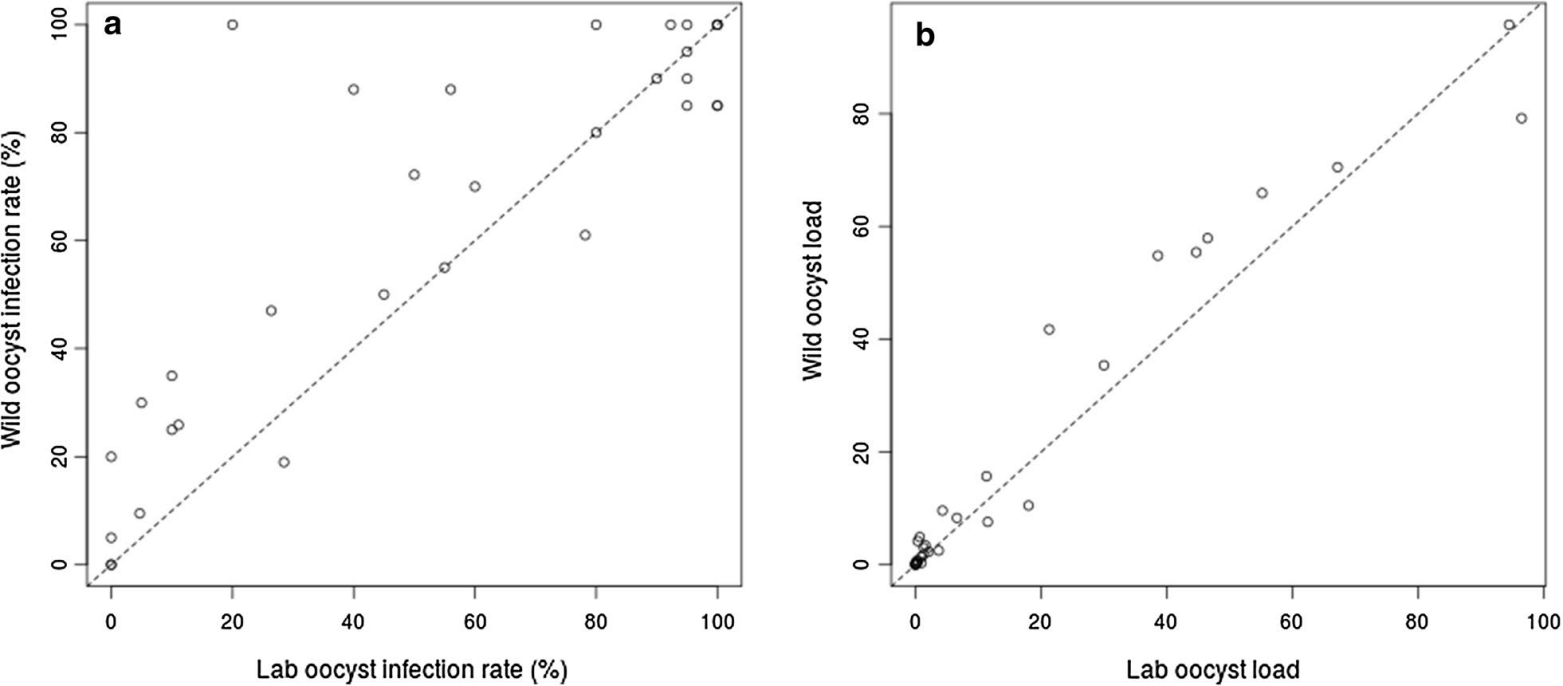
Oocyst infection rate and average oocyst load in laboratory and wild *Anopheles stephensi*. There is significant difference in **a** oocyst infection rate (paired t test, p = 0.01) and **b** no difference in average oocyst load (paired t test, p = 0.06) between wild and laboratory *An. stephensi*

Studying the development of oocysts in mosquitoes from different sources was expected to reveal potential differences in ability to support development of different patient isolates of *P. vivax*. The oocyst load in 32 wild *versus* colonized feeding experiments are shown in Table 1. Oocyst infection rate ranged from 0 to 100% in both wild and colonized mosquitoes, with mean (SD) 62.8% (35.2) and 53.8% (39.0) in wild and colonized mosquitoes, respectively. The 25 percentile, median and 75 percentile oocyst infection rate in wild (laboratory) mosquitoes was 29.0% (10.8%), 76.1% (55.5%) and 91.2% (95%), respectively. This means that 25% of wild samples have oocyst infection rate lower than 29.0, 25% of colonized samples have oocyst infection rate lower than 10.8%. Similarly, 50% of wild samples have oocyst infection rate lower than 76.1 and 50% of colonized samples have oocyst infection rate lower than 55.5%. Finally, 75% of wild samples have oocyst infection rate lower than 91.2 and 75% of colonized samples have oocyst infection rate lower than 95%. The oocyst load ranged from 0 to 215 in the wild and 0–210 in the colonized populations. The 25 percentile, median and 75 percentile average oocyst load in wild (laboratory) was 0.5 (0.3), 3.8 (1.9) and 37.0 (23.5), respectively. Statistically, there is significant difference between wild and colonized *An. stephensi* in the oocyst infection rate (paired t test, p = 0.01, mean of the differences = 9.0%) and no difference in the average oocyst load (paired t test, p = 0.06, mean of the differences = 2.3) (Fig. 1a, b). These comparative feeding experiments reveal a significant difference between wild and colonized mosquitoes in their susceptibility to oocyst stage infection of *P. vivax*.

**Table 1:** Oocyte infection rate and load in wild and colonized *Anopheles stephensi*

Experiment no.	Gametocytaemia	Wild mosquitoes					Colonized mosquitoes				
		No. dissected	No. positive	Oocyst range	Average oocyst no.	Oocyst infection rate (%)	No. dissected	No. positive	Oocyst range	Average oocyst no.	Oocyst infection rate (%)
1	0.03	27	7	1–3	0.33	25.9	27	3	1–4	0.29	11.1
2	0.56	23	14	0–13	2.5	61	23	18	0–14	3.7	78.2
3	0.09	26	26	4–195	65.9	100	26	24	0–175	55.2	92.3
4	0.04	34	16	0–83	4.2	47	34	9	0–3	0.35	26.4
5	0.13	25	22	0–15	4.9	88	25	10	0–5	0.68	40
6	0.58	20	17	0–215	95.8	85	20	20	25–210	94.5	100
7	0.23	20	18	0–40	15.7	90	20	18	0–30	11.3	90
8	0.18	20	17	0–185	79.2	85	20	20	10–192	96.5	100
9	0.05	20	18	0–22	7.6	90	20	19	0–24	11.5	95
10	0.14	20	16	0–50	10.5	80	20	16	0–65	18	80
11	0.31	20	4	0–1	0.2	20	20	0	0–0	0	0
12	0.19	21	2	0–2	0.14	9.5	21	1	1	0.05	4.7
13	0.15	20	7	0–4	0.75	35	20	2	0–4	0.25	10
14	0.16	20	14	0–8	2.3	70	20	12	0–15	2.1	60
15	0.06	20	6	0–2	0.35	30	20	1	1	0.05	5
16	0.11	20	17	0–85	35.4	85	20	19	0–68	30	95
17	0.14	20	20	22–102	54.8	100	20	19	0–75	38.6	95
18	0.05	20	10	0–9	1.8	50	20	9	0–5	1.3	45
19	0.02	21	4	0–3	0.3	19	21	6	0–12	0.95	28.5
20	0.03	16	0	0	0	0	16	0	0	0	0
21	0.06	5	5	1–9	3.4	100	5	1	8	1.6	20
22	0.09	18	13	0–12	3	72.2	18	9	0–7	1.3	50
23	0.04	14	0	0	0	0	14	0	0	0	0
24	0.03	25	22	0–35	9.6	88	25	14	0–22	4.3	56
25	0.08	20	11	0-8	1.4	55	20	11	0–3	0.95	55
26	0.14	20	20	1–127	41.7	100	20	16	0–55	21.3	80
27	0.12	20	1	1	0.05	5	20	0	0	0	0
28	0.17	12	12	21–105	57.9	100	12	12	1–85	46.5	100
29	0.29	20	5	0–4	0.55	25	20	2	0–5	0.35	10
30	0.27	17	17	13–165	55.4	100	17	17	4–89	44.7	100
31	0.09	20	19	0–42	8.3	95	20	19	0–25	6.6	95
32	0.3	15	15	21–145	70.5	100	15	15	1–132	67.2	100

The data from experiments 1–15 in wild mosquitoes were used in Balabaskaran et al. [23] to study oocyst infection kinetics in wild mosquitoes

### Sporozoite infection in wild and colonized *Anopheles stephensi*

**Fig. 2:**
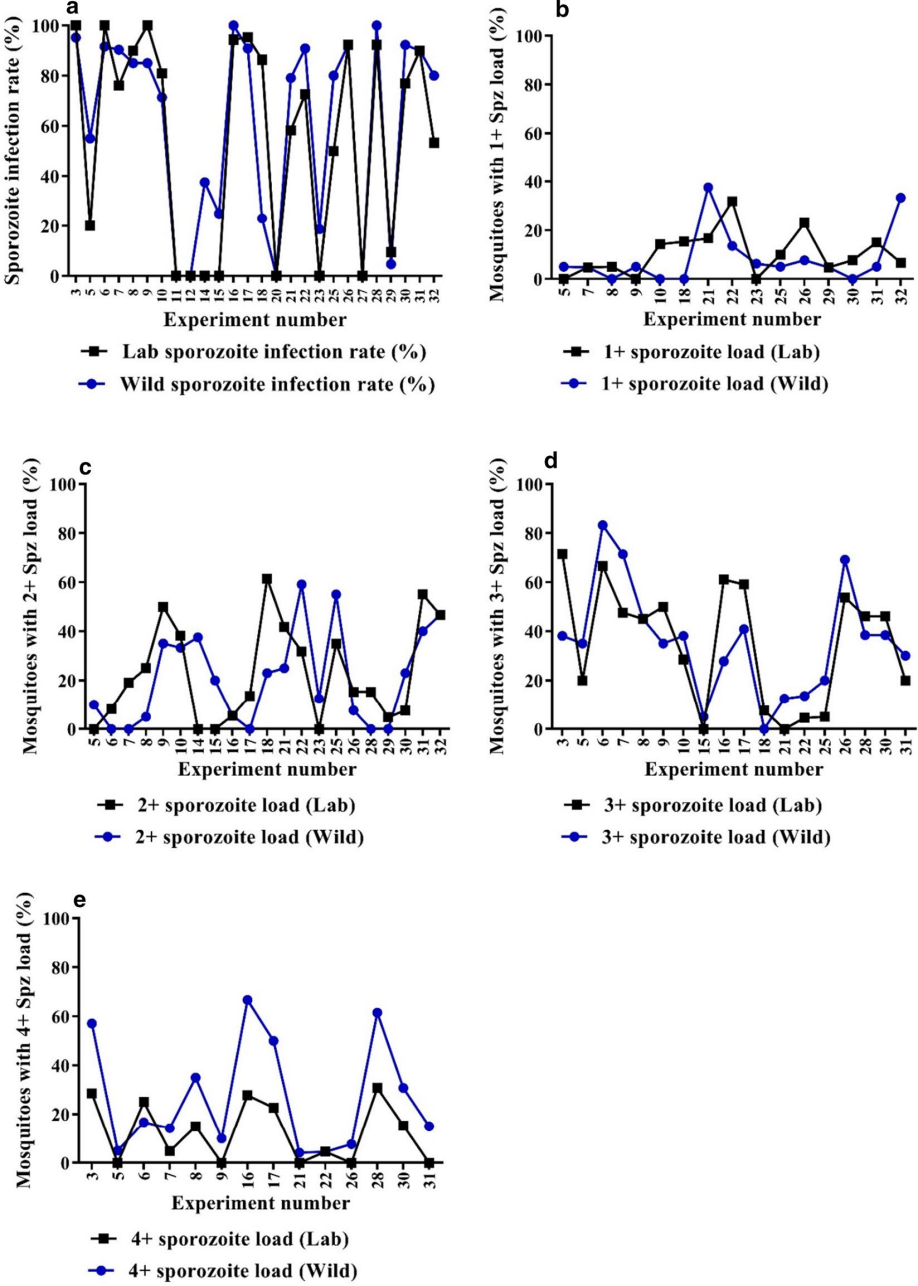
Sporozoite infection rate and 4+ sporozoite load in laboratory and wild *Anopheles stephensi*. **a** There is no significant difference in the sporozoite infection rate (paired t test, p = 0.18). **b**, **c**, **d** There is no significant difference in 1+, 2+ and 3+ sporozoite load; paired t test, p = 0.67, 0.53 and 0.89 respectively. **e** There is a significant difference in 4+ sporozoite load (paired t test, p = 0.002) between wild and laboratory *An. stephensi*. The values on Y axis were normalized and is reported as percent (%). Experiment number of each comparison experiment is plotted in the x axis

Sporozoite infection rate and sporozoite load was expected to reveal possible variations in the infection kinetics of patient-derived isolates of *P. vivax* in wild *versus* colonized *An. stephensi* and potential variations in transmission potential of different *P. vivax* samples. In 26 of the 32 experiments, there were surviving laboratory and wild mosquitoes on days 12 and 13, and these were dissected to assess sporozoite infection rate and gland index. The details of sporozoite infection rate and load of individual experiments are given in Table 2. The sporozoite infection rate ranged from 0 to 100% in wild and colonized mosquitoes, and the mean (SD) was 60.7 (38.0)% and 55.3 (40.9)% in wild and colonized respectively. The 25 percentile, median and 75 percentile oocyst infection rate in wild and (laboratory) was 23.5 (2.4), 80 (74.4) and 90.9 (91.7), respectively. In experiments 14, 15 and 23 (Table 2), sporozoites were not seen in the colonized mosquitoes, whereas the infection rate in wild mosquitos was 37.5, 25 and 18.75%, respectively. There was no significant difference (paired t test, p = 0.18) in the sporozoite infection rate in wild and colonized mosquitoes (Fig. 2a). Both wild and colonized mosquitoes had a wide range of sporozoite load ranging from 1+ to 4+. The number of mosquitoes with gland index of 1+, 2+ and 3+ were similar in wild and colonized (Table 3), and the two types of mosquitoes showed no significant difference; paired t test p value is 0.67, 0.53, 0.89 for 1+, 2+ and 3+ mosquitoes, respectively (Fig. 2b–d). Interestingly, in 12 of the 14 experiments where mosquitoes with 4+ gland index were seen (wild or colonized), the number of mosquitoes with 4+ sporozoite load were higher in wild (68) than in colonized mosquitoes (30) (Table 3), and the difference is significant (paired t test, p = 0.002) (Fig. 2e). Overall, the sporozoite infection rate in wild and colonized mosquitoes may be similar, however, the parasites reach high sporozoite levels more efficiently in wild mosquitoes compared to colonized mosquitoes.

**Table 2:** Sporozoite infection rate and load in wild and colonized *An. stephensi*

Experiment no.	Gametocytaemia	Wild mosquitoes				Colonized mosquitoes			
		No. dissected	No. positive	Sporozoite infection rate (%)	Gland index	No. dissected	No. positive	Sporozoite infection rate (%)	Gland index
3	0.097	21	20	95.2	4+ (12), 3+ (8)	21	21	100	4+ (6), 3+ (15)
5	0.136	20	11	55	4+ (1), 3+ (7), 2+ (2), 1+ (1)	20	4	20	3+ (4)
6	0.585	12	11	91.6	4+ (2), 3+ (10)	12	12	100	4+ (3), 3+ (8), 2+ (1)
7	0.235	21	19	90.4	4+ (3), 3+ (15), 1+ (1)	21	16	76.1	4+ (1), 3+ (10), 2+ (4), 1+ (1)
8	0.18	20	17	85	4+ (7), 3+ (9), 2+ (1)	20	18	90	4+ (3), 3+ (9), 2+ (5), 1+ (1)
9	0.058	20	17	85	4+ (2), 3+ (7), 2+ (7), 1+ (1)	20	20	100	3+ (10), 2+ (10)
10	0.143	21	15	71.4	3+ (8), 2+ (7)	21	17	80.9	3+ (6), 2+ (8), 1+ (3)
11	0.3195	4	0	0	–	4	0	0	–
12	0.198	20	0	0	–	20	0	0	–
14	0.16	8	3	37.5	2+ (3)	8	0	0	–
15	0.06	20	5	25	3+ (1), 2+ (4)	20	0	0	–
16	0.115	18	18	100	4+ (12), 3+ (5), 2+ (1)	18	17	94.4	4+ (5), 3+ (11), 2+ (1)
17	0.145	22	20	90.9	4+ (11), 3+ (9)	22	21	95.4	4+ (5), 3+ (13), 2+ (3)
18	0.055	13	3	23	2+ (3)	13	11	84.6	3+ (1), 2+ (8), 1+ (2)
20	0.037	8	0	0	–	8	0	0	–
21	0.0625	24	19	79.1	4+ (1), 3+ (3), 2+ (6), 1+ (9)	24	14	58.3	2+ (10), 1+ (4)
22	0.095	22	20	90.9	4+ (1), 3+ (3), 2+ (13), 1+ (3)	22	16	72.7	4+ (1), 3+ (1), 2+ (7), 1+ (7)
23	0.040	16	3	18.7	2+ (2), 1+ (1)	16	0	0	0
25	0.088	20	16	80	3+ (4), 2+ (11), 1+ (1),	20	10	50	3+ (1), 2+ (7), 1+ (2)
26	0.141	13	12	92.3	4+ (1), 3+ (9), 2+ (1), 1+ (1)	13	12	92.3	3+ (7), 2+ (2), 1+ (3)
27	0.122	11	0	0	0	11	0	0	0
28	0.176	13	13	100	4+ (8), 3+ (5)	13	12	92.3	4+ (4), 3+ (6), 2+ (2)
29	0.295	21	1	4.7	1+ (1)	21	2	9.5	2+ (1), 1+ (1)
30	0.274	13	12	92.3	4+ (4), 3+ (5), 2+ (3)	13	10	76.9	4+ (2), 3+ (6), 2+ (1), 1+ (1)
31	0.092	20	18	90	4+ (3), 3+ (6), 2+ (8), 1+ (1)	20	18	90	3+ (4), 2+ (11), 1+ (3),
32	0.3	15	12	80	2+ (7), 1+ (5)	15	8	53.3	2+ (7), 1+ (1)

Experiment numbers in Table 2 correspond to experiment numbers in Table 1. The data from experiments 3, 5, 6–12, 14 and 15 in wild mosquitoes were used in Balabaskaran et al. [23] to study sporozoite infection kinetics in wild mosquitoes

**Table 3:** Distribution of sporozoite load in wild and colonized mosquitoes

Sporozoite load (gland index)	Wild mosquitoes	Colonized mosquitoes
1+	25	29
2+	79	88
3+	112	114
4+	68	30

Experiment numbers in Table 2 correspond to experiment numbers in Table 1. The data from experiments 3, 5, 6–12, 14 and 15 in wild mosquitoes were used in Balabaskaran et al. [23] to study sporozoite infection kinetics in wild mosquitoes.

## Discussion

Colonized mosquitoes are widely used around the world to understand the developmental kinetics of *Plasmodium* isolates of that region [1–4, 8, 22, 25, 26]. Upon colonization, mosquitoes tend to undergo genetic variation over a period of many generations [14, 17, 18]. How this genetic diversity associated with mosquito colonization affects *Plasmodium* development has not been clear. Studies have mapped gene polymorphisms to altered levels of vector susceptibility to *Plasmodium* [5, 11, 27, 28]. *Anopheles gambiae* from different geographical locations in Africa show different infection intensity to infection with *Plasmodium falciparum* [29], underscoring the importance of genetic differentiation. Even though varying infection intensities has been reported between sympatric and allopatric vector-parasite combinations [29] or between specific intercrosses of mosquito phenotypes [5], the effect of genetic differentiation on highly passaged colonized mosquitoes to *Plasmodium* infection, especially *P. vivax* infection, has not been investigated so far.

Mosquito infection experiments in wild Anophelines are complicated by logistics, availability of mosquitoes year around, their ability to adapt to artificial feeding and new environment. The MESA-ICEMR mosquito infection laboratory at NIMR-Goa is one of the very few sites in the world where feeding experiments can be done with wild *An. stephensi* and patient isolates of *P. vivax*. The presence of highly passaged *An. stephensi* (66th–86th generations) allowed to study the susceptibility of wild and colonized mosquitoes to patient derived isolates of *P. vivax*. In both the mosquito populations, there is significant difference in oocyst infection rate and whereas this was not in the average oocyst load. In three of the experiments, no sporozoites were found in the laboratory when compared to wild mosquitoes. The average oocyst load ranged between 0 and 2.5 in these experiments, and is possible that some parasite strains may not develop to sporozoites as efficiently in wild as compared to laboratory, especially when the oocyst load is very low. When the sporozoite load was compared between wild and laboratory, there was no significant difference in 1+, 2+ and 3+ load. However, in 12 of the 14 experiments where 4+ sporozoite load were seen, the number of mosquitoes with 4+ load was significantly higher in wild (68) than in colonized (30) mosquitoes. In *An. gambiae*, it has been shown that genes involved in insecticide resistance, immunity and olfaction are expressed higher in wild mosquitoes when compared to colonized populations [14]. Hence, it is important to learn here that, even under sterile rearing conditions lacking continual parasite infections, the immune system in colonized *An. stephensi* was not weakened when challenged with *P. vivax*. It is still possible that, in colonized mosquitoes, there could be a developmental delay that prevents the sporozoites to populate the salivary gland on days 12 and 13.

Mosquito gut microbiota is determined by the water source available in the breeding habitats, and has been shown to modulate the development of *P. falciparum* [30–37]. Anti-*Plasmodium* effect of gut microbiota is suggested to be due to the effect of bacterial compounds and/or mosquito immunity directed against the microbes [31, 38]. It is possible that the altered gut biota in colonized mosquitoes may elicit stronger basal immunity than in wild, and produce metabolites that may affect oocyst maturation and sporozoite development. A recent study has implicated a specific *Escherichia coli* strain 444_ST95_ in modulating *P. falciparum* infection in the mosquito midgut [38]. The observed differences in 4+ sporozoite load between wild and colonized mosquitoes may be further investigated in the future.

Overall, at the level of oocyst development, significant difference was found between the colonized and wild mosquitoes in their susceptibility to *P. vivax*. To the first approximation, it should be possible to use freshly reared larvae of other mosquito species to understand parasite infectivity. Although there was no significant difference in sporozoite infection rate in the present study, a significantly higher sporozoite load (4+) was found in wild when compared to laboratory mosquitoes. These experiments illustrate why it may be important to exercise caution when studying parasite infection in long-term laboratory-reared colonies of *An. stephensi*, especially in this region. It will be of interest to learn whether long-term mosquito colonization alters *Plasmodium* susceptibility in vectors from different geographical locations. Understanding the molecular mechanisms that modulate *Plasmodium* infection will also be a prime area of focus in the future studies.

## Abbreviations

*An*: *Anopheles*
GMC: Goa Medical College and Hospital
MESA: malaria evolution in South Asia
ICEMR: International Center of Excellence for Malaria Research
NIMR: National Institute of Malaria Research
NIAID: National Institute of Allergy and Infectious Diseases
NIH: US National Institutes of Health
US: United States of America
UW: University of Washington
DMID: Division of Microbiology and Infectious Disease

## References

[1] Zhu G, Xia H, Zhou H, Li J, Lu F, Liu Y (2013). Susceptibility of *Anopheles sinensis* to *Plasmodium vivax* in malarial outbreak areas of central China. Parasit Vectors.

[2] Zollner GE, Ponsa N, Garman GW, Poudel S, Bell JA, Sattabongkot J (2006). Population dynamics of sporogony for *Plasmodium vivax* parasites from western Thailand developing within three species of colonized Anopheles mosquitoes. Malar J.

[3] Thongsahuan S, Baimai V, Junkum A, Saeung A, Min GS, Joshi D (2011). Susceptibility of *Anopheles campestris*-like and *Anopheles barbirostris* species complexes to *Plasmodium falciparum* and *Plasmodium vivax* in Thailand. Mem Inst Oswaldo Cruz.

[4] Joshi D, Choochote W, Park MH, Kim JY, Kim TS, Suwonkerd W (2009). The susceptibility of *Anopheles lesteri* to infection with Korean strain of *Plasmodium vivax*. Malar J.

[5] White BJ, Lawniczak MK, Cheng C, Coulibaly MB, Wilson MD, Sagnon N (2011). Adaptive divergence between incipient species of *Anopheles gambiae* increases resistance to *Plasmodium*. Proc Natl Acad Sci USA.

[6] Tchuinkam T, Mulder B, Dechering K, Stoffels H, Verhave JP, Cot M (1993). Experimental infections of *Anopheles gambiae* with *Plasmodium falciparum* of naturally infected gametocyte carriers in Cameroon: factors influencing the infectivity to mosquitoes. Trop Med Parasitol.

[7] Rios-Velasquez CM, Martins-Campos KM, Simoes RC, Izzo T, dos Santos EV, Pessoa FA (2013). Experimental *Plasmodium vivax* infection of key *Anopheles* species from the Brazilian Amazon. Malar J.

[8] Moreno M, Tong C, Guzman M, Chuquiyauri R, Llanos-Cuentas A, Rodriguez H (2014). Infection of laboratory-colonized *Anopheles darlingi* mosquitoes by *Plasmodium vivax*. Am J Trop Med Hyg.

[9] Gonzalez-Ceron L, Rodriguez MH, Nettel JC, Villarreal C, Kain KC, Hernandez JE (1999). Differential susceptibilities of *Anopheles albimanus* and *Anopheles pseudopunctipennis* to infections with coindigenous *Plasmodium vivax* variants VK210 and VK247 in southern Mexico. Infect Immun.

[10] Vallejo AF, Rubiano K, Amado A, Krystosik AR, Herrera S, Arevalo-Herrera M (2016). Optimization of a membrane feeding assay for *Plasmodium vivax* infection in *Anopheles albimanus*. PLoS Negl Trop Dis.

[11] Collins FH, Sakai RK, Vernick KD, Paskewitz S, Seeley DC, Miller LH (1986). Genetic selection of a *Plasmodium*-refractory strain of the malaria vector *Anopheles gambiae*. Science.

[12] Vernick KD, Fujioka H, Seeley DC, Tandler B, Aikawa M, Miller LH (1995). *Plasmodium gallinaceum*: a refractory mechanism of ookinete killing in the mosquito, *Anopheles gambiae*. Exp Parasitol.

[13] Muller P, Donnelly MJ, Ranson H (2007). Transcription profiling of a recently colonised pyrethroid resistant *Anopheles gambiae* strain from Ghana. BMC Genomics.

[14] Aguilar R, Simard F, Kamdem C, Shields T, Glass GE, Garver LS (2010). Genome-wide analysis of transcriptomic divergence between laboratory colony and field *Anopheles gambiae* mosquitoes of the M and S molecular forms. Insect Mol Biol.

[15] Hoffmann AA, Hallas R, Sinclair C, Partridge L (2001). Rapid loss of stress resistance in *Drosophila melanogaster* under adaptation to laboratory culture. Evolution.

[16] Reed DH, Lowe EH, Briscoe DA, Frankham R (2003). Fitness and adaptation in a novel environment: effect of inbreeding, prior environment, and lineage. Evolution.

[17] Norris DE, Shurtleff AC, Toure YT, Lanzaro GC (2001). Microsatellite DNA polymorphism and heterozygosity among field and laboratory populations of *Anopheles gambiae* ss (Diptera: Culicidae). J Med Entomol.

[18] Lainhart W, Bickersmith SA, Moreno M, Rios CT, Vinetz JM, Conn JE (2015). Changes in genetic diversity from field to laboratory during colonization of *Anopheles darlingi* root (Diptera: Culicidae). Am J Trop Med Hyg.

[19] Kumar A, Hosmani R, Jadhav S, de Sousa T, Mohanty A, Naik M (2016). *Anopheles subpictus* carry human malaria parasites in an urban area of Western India and may facilitate perennial malaria transmission. Malar J.

[20] Nagpal B, Sharma V (1995). Indian anophelines.

[21] Christophers S (1933). The Fauna of British India, including Ceylon and Burma. Diptera family Culicidae tribe Anophelini.

[22] Solarte Y, Manzano MR, Rocha L, Hurtado H, James MA, Arevalo-Herrera M (2011). *Plasmodium vivax* sporozoite production in *Anopheles albimanus* mosquitoes for vaccine clinical trials. Am J Trop Med Hyg.

[23] Balabaskaran Nina P, Mohanty AK, Ballav S, Vernekar S, Bhinge S, D’Souza M (2017). Dynamics of *Plasmodium vivax* sporogony in wild *Anopheles stephensi* in a malaria-endemic region of Western India. Malar J.

[24] Riley RS, Ben-Ezra JM, Goel R, Tidwell A (2001). Reticulocytes and reticulocyte enumeration. J Clin Lab Anal.

[25] Vallejo AF, Garcia J, Amado-Garavito AB, Arevalo-Herrera M, Herrera S (2016). *Plasmodium vivax* gametocyte infectivity in sub-microscopic infections. Malar J.

[26] Basseri HR, Doosti S, Akbarzadeh K, Nateghpour M, Whitten MM, Ladoni H (2008). Competency of *Anopheles stephensi mysorensis* strain for *Plasmodium vivax* and the role of inhibitory carbohydrates to block its sporogonic cycle. Malar J.

[27] Blandin SA, Wang-Sattler R, Lamacchia M, Gagneur J, Lycett G, Ning Y (2009). Dissecting the genetic basis of resistance to malaria parasites in *Anopheles gambiae*. Science.

[28] Vernick KD, Oduol F, Lazzaro BP, Glazebrook J, Xu J, Riehle M (2005). Molecular genetics of mosquito resistance to malaria parasites. Curr Top Microbiol Immunol.

[29] Harris C, Morlais I, Churcher TS, Awono-Ambene P, Gouagna LC, Dabire RK (2012). *Plasmodium falciparum* produce lower infection intensities in local versus foreign *Anopheles gambiae* populations. PLoS ONE.

[30] Boissiere A, Tchioffo MT, Bachar D, Abate L, Marie A, Nsango SE (2012). Midgut microbiota of the malaria mosquito vector *Anopheles gambiae* and interactions with *Plasmodium falciparum* infection. PLoS Pathog.

[31] Dong Y, Manfredini F, Dimopoulos G (2009). Implication of the mosquito midgut microbiota in the defense against malaria parasites. PLoS Pathog.

[32] Cirimotich CM, Dong Y, Clayton AM, Sandiford SL, Souza-Neto JA, Mulenga M (2011). Natural microbe-mediated refractoriness to *Plasmodium* infection in *Anopheles gambiae*. Science.

[33] Pumpuni CB, Beier MS, Nataro JP, Guers LD, Davis JR (1993). *Plasmodium falciparum*: inhibition of sporogonic development in *Anopheles stephensi* by gram-negative bacteria. Exp Parasitol.

[34] Tchioffo MT, Boissiere A, Churcher TS, Abate L, Gimonneau G, Nsango SE (2013). Modulation of malaria infection in *Anopheles gambiae* mosquitoes exposed to natural midgut bacteria. PLoS ONE.

[35] Beier MS, Pumpuni CB, Beier JC, Davis JR (1994). Effects of para-aminobenzoic acid, insulin, and gentamicin on *Plasmodium falciparum* development in anopheline mosquitoes (Diptera: Culicidae). J Med Entomol.

[36] Wang S, Dos-Santos ALA, Huang W, Liu KC, Oshaghi MA, Wei G (2017). Driving mosquito refractoriness to *Plasmodium falciparum* with engineered symbiotic bacteria. Science.

[37] Sharma A, Dhayal D, Singh OP, Adak T, Bhatnagar RK (2013). Gut microbes influence fitness and malaria transmission potential of Asian malaria vector *Anopheles stephensi*. Acta Trop.

[38] Tchioffo MT, Abate L, Boissiere A, Nsango SE, Gimonneau G, Berry A (2016). An epidemiologically successful *Escherichia coli* sequence type modulates *Plasmodium falciparum* infection in the mosquito midgut. Infect Genet Evol.

